# Are we ready for the next anthrax outbreak? Lessons from a simulation exercise in a rural-based district in Uganda

**DOI:** 10.1017/S0950268824001493

**Published:** 2024-12-02

**Authors:** Abel W. Walekhwa, Lydia N. Namakula, Brenda Nakazibwe, Richard Ssekitoleko, Lawrence Mugisha

**Affiliations:** 1 IDEMU Mathematical Modelling Unit, Kampala, Uganda; 2 Science, Technology and Innovation, Secretariat—Office of the President, Kampala, Uganda; 3 School of Public Health, College of Health Sciences, Makerere University, Kampala, Uganda; 4 World Health Organisation, Uganda Country office, Kampala, Uganda; 5 Department of Wildlife, Animal Resources Management, College of Veterinary Medicine, Animal Resources and Biosecurity, Makerere University, Kampala, Uganda

**Keywords:** anthrax outbreak, preparedness, simulation, surveillance, Uganda

## Abstract

Anthrax is a bacterial zoonotic disease caused by *Bacillus anthracis.* We qualitatively examined facilitators and barriers to responding to a potential anthrax outbreak using the capability, opportunity, motivation behaviour model (COM-B model) in the high-risk rural district of Namisindwa, in Eastern Uganda. We chose the COM-B model because it provides a systematic approach for selecting evidence-based techniques and approaches for promoting the behavioural prompt response to anthrax outbreaks. Unpacking these facilitators and barriers enables the leaders and community members to understand existing resources and gaps so that they can leverage them for future anthrax outbreaks.

This was a qualitative cross-sectional study that was part of a bigger anthrax outbreak simulation study conducted in September 2023. We conducted 10 Key Informant interviews among key stakeholders. The interviews were audio recorded on Android-enabled phones and later transcribed verbatim. The transcripts were analyzed using a deductive thematic content approach through Nvivo 12.

The facilitators were; knowledge of respondents about anthrax disease and anthrax outbreak response, experience and presence of surveillance guidelines, availability of resources, and presence of communication channels. The identified barriers were; porous boarders that facilitate unregulated animal trade across, lack of essential personal protective equipment, and lack of funds for surveillance and response activities.

Generally, the district was partially ready for the next anthrax outbreak. The district was resourced in terms of human resources but lacked adequate funds for animal, environmental and human surveillance activities for anthrax and related response. The district technical staff had the knowledge required to respond to the anthrax outbreak but lacked adequate funds for animal, environmental and human surveillance for anthrax and related response. We think that our study findings are generalizable in similar settings and therefore call for the implementation of such periodic evaluations to help leverage the strong areas and improve other aspects. Anthrax is a growing threat in the region, and there should be proactive efforts in prevention, specifically, we recommend vaccination of livestock and further research for human vaccines.

## Introduction

The World Health Organization (WHO) defines anthrax as an infection caused by the spore-forming bacteria, *Bacillus anthracis.* It is a zoonosis (disease transmissible from animals to humans) that typically affects ruminants (such as cows, sheep, and goats). The bacteria produce extremely potent toxins which are responsible for the symptoms, causing high morbidity. Humans are infected with anthrax from infected animals or through contaminated animal products. The WHO estimates that about 95% of the anthrax cases are cutaneous resulting from handling infected carcasses or the hides, hair, meat, or bones from such carcasses [1]. Different epidemiological studies have demonstrated that due to ecological suitability but also the potential of the virus staying for longer periods in the soil there is a growing threat of anthrax in sub-Saharan Africa [2–4]. The main predisposing factors for the continued spread of anthrax are human behaviors such as not using personal protective equipment during slaughter or meat handling practices [3]. Currently, the vaccine for anthrax exists but few countries have an animal vaccination programme as the disease is considered a public good [5].

In Sub-Saharan Africa, there is passive surveillance with the possibility of the disease spreading without being detected [6]. But also given the trade and tourism consequences/implications that emerge if the disease is confirmed, some countries prefer not to report during sporadic outbreaks. Ndolo et al. [2] conducted an ecological niche model for Uganda and Kenya and showed key hotspots for anthrax in the two countries. Specifically, in Uganda since the risk map was developed, five independent outbreaks have been declared by the Ministry of Health, Uganda. However, these outbreaks take the country by surprise and may interrupt economic activities specifically the production activities among livestock [7, 8].

This study is part of the bigger project where we hypothesized that Uganda as a country may not be ready for the next anthrax outbreak [9]. In this project, we aimed to pursue risk assessment through a qualitative approach with Namisindwa district as a case study. Risk assessments are documented as part of the strategies that will enable countries to get prepared for the next pandemic and are specified in the National Action Plan for Health Security (NAPHS) [10]. In this study, we aimed to document the capabilities, opportunities motivations, and related barriers for a rural-based district in responding to a potential anthrax disease in an ideal time (when there is no confirmed anthrax outbreak).

We chose the COM-B model because it has successfully been applied to study behavioural-related interventions similar to what we wanted to explore [11–13]. The intervention for our case was the two days training which we gave to the stakeholders who were interviewed. The detailed description of the intervention is documented in a sister study by [9]. We also wanted to explore a holistic understanding of the factors influencing the behaviour of responding to potential outbreaks in this area. Following this motivation, we thought that understanding the barriers and facilitators would inform policymakers at different levels of what we need to do to prepare for future outbreaks. However, unpacking these facilitators enables the leaders and community members to understand what available resources they could leverage for future outbreaks. This COM-B model also provides a systematic approach for selecting evidence-based techniques and approaches to promote the behaviour of early preparedness for potential anthrax outbreaks [13, 14].

## Materials and methods

### Study site

As described earlier this was part of a bigger study by [9] and it was conducted in Namisindwa District, Eastern Uganda [9]. The bigger study was a simulation exercise that involved sending a message alert of ongoing animal deaths in Namisindwa district with a description of anthrax signs. This message was sent to the WhatsApp groups of human and animal health professionals, and members of the Task Forces at the district level and the level of the Ministry of Health. The message was sent by a reliable individual from the Ministry of Health. Meanwhile, the research team was at the district headquarters ready to interview key informants about their knowledge of anthrax, preparedness, response and influencing factors.

Namisindwa district is bordered by Bududa District to the north, Kenya to the east and south, Tororo District to the south-west, and Manafwa District to the west. Neighbouring districts such as Buduuda experienced their first-ever anthrax outbreak in 2022 [15]. This outbreak resulted in the quarantine of the animal population in neighbouring districts including the Manafwa and Namisindwa districts [16,17]. Anthrax outbreaks have also occurred along the border districts of Western Kenya, which borders Namisindwa district as well [18]. This makes Namisindwa district a suitable site for the study.

**Figure 1.**
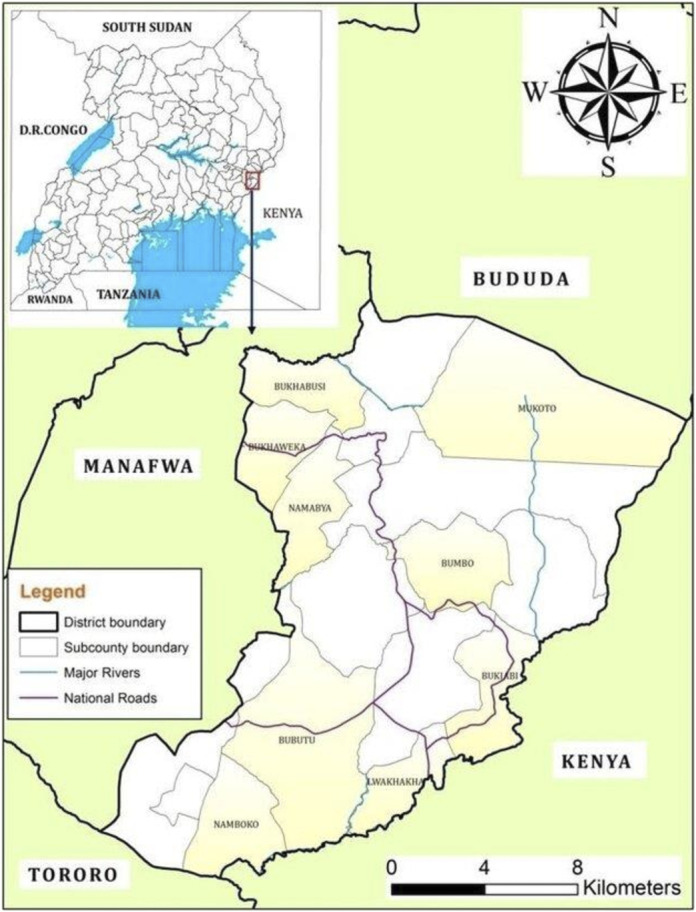
Map of Uganda showing the location of Namisindwa District (Ongom et al., 2020).

The district headquarters at Bupoto is located approximately 40 km (25 miles) by road, southeast of Mbale, the largest city of the sub-region. The coordinates of the district are 00°49′N 34°23′E. The people in this district practice small-scale agriculture for their livelihood and rear different livestock, including cattle, goats, sheep, and donkeys. This district has multiple porous border points with Kenya, which promotes the illegal movement of cattle from Kenya into Uganda (Figure 1), making them susceptible to zoonotic disease outbreaks (Figure 1).

### Study design and data collection

A cross-sectional study using qualitative data collection methods was designed for this project and data was collected in September 2023. The study participants were professionals who identified as males and females employed by the government of Uganda and charged with disease outbreaks and responses. They were chosen because anthrax is one of the diseases listed among the priority diseases in Uganda and this requires response and notification whenever there is a suspected outbreak [19].

We conducted ten (10) Key Informant Interviews (KII) (five male and five female) with the District Taskforce (DTF) and District Rapid Response Team (DRRT) members using a pretested KII guide. Additional qualitative data was collected during dissemination meetings with district task force members after the simulation. The interview guide was composed of open-ended questions and probes as per requirement. Socio-demographic questions such as age, gender, designation, and years in service were also included. This was aimed at understanding the study participants better. All the interviews were audio recorded after obtaining the consent of the study participants. An audio recorder was used for recording the interviews and each interview lasted about 30–40 min. All the audio recordings from KII were transcribed verbatim and analyzed through a deductive thematic content approach. We then read through all the text of the documents and categorized them into different themes capabilities, Opportunities, and Motivation. Initially, meaning units were extracted from original transcripts and condensed to form codes. The meaning units which were selected were linked to the aim of the study. Related codes were grouped to make themes which demonstrated the core meanings of the texts were identified. Furthermore, we categorized codes from the text into relevant subthemes under each main theme. We imported the transcribed KII documents into Nvivo 12 for qualitative analysis. In the process of the analysis, the first columns of the codebook were designed into the theme column with a description of each theme and potential examples for easy allocation of texts. After coding all the text, we completed the code book into the following columns; theme, subtheme, description, number of files, number of references, and quotation. This was conducted in Ms. Excel 2019. This was finally followed by a report in Ms. Word highlighting the main generated themes, subthemes, and quotations.

### Ethical considerations

We obtained Institutional Review Board (IRB) approval from Makerere University School of Public Health (SPH-2023-463) and the protocol was registered with the Uganda National Council for Science and Technology (HS3136ES). In addition, administrative clearance from Namisindwa district police station was obtained as a process of district entry for the planned activities. We translated the informed consent and data collection tools into the local language. This enabled smooth communication between the research team and all stakeholders. We worked with local leaders and obtained written informed consent and permission for all the participating teams from their organizations/workplaces during all the project activities.

We sought written informed consent from selected respondents before participating in the study. All participants were informed of their voluntary participation in the study and their freedom to withdraw from the study at any time.

## Results

A total of five (5) themes were generated from the data and these included Capability, Opportunities and Motivation which followed the COM-B model. The other two additional themes included barriers to readiness for anthrax outbreak response and Recommendations for improved anthrax outbreak response. A total of 15 subthemes were generated for the capabilities, opportunities and motivation themes, each of which was described with respective quotations. The subthemes for capability include Knowledge about anthrax and anthrax outbreak response, Experience and presence of surveillance guidelines. Subthemes for opportunities include Resources (such as human and financial resources), logistics, presence of communication channels and socio-economic activities in the district. Subthemes for motivation include beliefs in the ability to respond, fear of anthrax, intentions for disease prevention and personal drive/emotions. The subthemes for barriers to readiness for anthrax outbreak response and recommendations for improved anthrax outbreak response were also structured into the capabilities, opportunities and motivation. This section therefore describes the themes and subthemes with their quotations.

### Capabilities

#### Knowledgeable about anthrax disease

Generally, we found that One Health stakeholders were knowledgeable about anthrax and the response mechanisms, although their response to the alert did not reflect their actions. There was generally, a knowledge-do gap. Some key informants knew the cause and types of anthrax, the signs and symptoms of anthrax, communication lines in case of an outbreak, and case management strategies, including the need to bury the dead carcass to prevent further transmission from the spores and communications lines/contacts in case of an outbreak (Table 1).

**Table 1.** Summary of knowledge about Anthrax disease

Subtheme: Knowledgeable about anthrax	Quotations
Cause of anthrax	‘…and it is a bacterial disease caused by *Bacillus anthracis.* And what I know with it it’s spores, when they are in soil, they can stay for longer time.’ Male KI 4 ‘This disease occurs when a cow presents with the appearance of blood, saliva-laden bubbles and dung mixed with blood.’ Male KI 6
Signs and symptoms	‘And I know anthrax is a zoonotic disease, we share it with the animals. And mostly when you eat the meat, which is infected, you get that disease. Or the milk or the products of the animal you get that disease.’ KI 1
Response towards anthrax	‘Why we even prefer burying is because when we dig that deep hole, we know because it has those spores and they’re resistant to heat. They stay for long. So, why sometimes it comes up, it’s because how people interact with the soil, with the soil around where they dug. Interact from there, the dust, sometimes they inhale, that inhalation.’ Female KI 5
Facilitators to knowledge; trainings	‘I’ve done surveillance for the last seven years. Since I was recruited, I’ve been trained in surveillance activities and I’m also a trained epidemiologist by background. So, I’ve been doing surveillance for the last seven years in this district.’ Male KI 2 ‘…‥As I told you that we recently had a training on IDSR [Integrated Diseases Surveillance Response], and that was also one of the components that the outbreaks were trained…’ Female KI 5

#### Knowledge about reporting approaches

The participants reported being informed about the stakeholders to call, warn or alert in case of a public health emergency such as an anthrax outbreak and were also informed about the responsibilities of these stakeholders.

We communicate to the Commissioner through Chief Administrative Officer (CAO) [of Namisindwa Distrct]. CAO is the one to communicate. We write to CAO, CAO is the one who communicates to the Commissioner. Female KI 4

Like, if it is in the community, you have to report to me [surveillance officer], then not reporting, but notifying me. Then I also notify the DHO [District Health Officer]. And we investigate the alert. And if we confirm, we must notify the center [Ministry of Health] and the Regional Emergency Operations Centre (EOC) immediately. And then we respond. Male KI 1

#### Experience of participants and training

Knowledge about the anthrax disease among the participants was attributed to their years of experience in zoonotic disease management, experience from previous outbreaks and the various training sessions such as surveillance and the experience in the area of outbreak response over the year.

As I told you that we recently had a training on IDSR [Integrated Diseases Surveillance Response], and that was also one of the components that the outbreaks were trained…IDSR. Male KI 5

I’ve done surveillance for the last seven years. Since I was recruited, I’ve been trained in surveillance activities and I’m also a trained epidemiologist by background. So, I’ve been doing surveillance for the last seven years in this district. Male KI 2

We succeeded because it [previous anthrax outbreak] didn’t spread further. By close of three months of quarantine we had contained and we didn’t lose many of the animals and meat was not being sold…. KI 1

#### Presence of surveillance guidelines

Participants reported the presence of established gazette areas for monitoring and designated marketplaces for animal trade and inspection.

we have some markets, designated markets. So, whenever these animals they are in the market, the vet guy inspects them and they give it permit before they send them to another place. KI 4

…all traders who go to Kenya. They have gazetted places, so they bring their animals, put them in a gazetted place and the sub county extension worker always goes and monitors. If he detects something, they inform [the district]. If he can manage, they can manage, they can manage it at that level. So, those are basically the safety precautions we put to ensure that we protect the livestock population in the district. KI 2

### Opportunities

#### Resources such as human and financial resource

Participants highlighted the presence of human resources such as district task force officers, district health and production departments, surveillance officers, technical staff, local leaders and implementing partners such as the Mbale Regional Public Health Emergency Operations Centre (RPHEOC) who work at different levels of Namisindwa district and Mbale region in support for outbreaks surveillance, prevention, and response. Participants also mentioned the presence of a budget that caters to public health emergencies.

First and foremost, it is a response team, it must have technical staff both on human and that’s on the DVO surveillance focal person, then DHO [District Health Officer] district Health officer with a surveillance focal person, with a lab officer, isn’t it? Because he is the one to pick the sample in a proper process. Then we also have the leadership, because in the leadership we have the RDC [Resident District Commissioner]. Sometimes we have the CAO himself. So, that is what is composed in that case. Male KI 3

We have both technical and the political at the sub-county, you know, these structures district sub-county to the village, we have those structures. So, if our vet guy at the sub-county level and then our environmental health staffs at the sub-county. So, these are the teams in case any information comes to the districts. We pass this information to these people and also they cascade it to the community. KI 4

Mbale EOC [Emergency Operations Centre] is very key because being the regional thing to the national, so it has to communicate actually the ministry and also the facilities. Male KI 5

as I told you by my office, Environmental Health division, that is where the district health also falls our mandate is to educate the public the preventive and control measures. Money? Yes, the budget is there and then we plan from the health center even the health center III, HCII they have the plan then do even up to the district. KI 4

#### Logistics

Participants mentioned the presence of logistics such as Information, Education and Communication (IEC) materials such as loudspeakers, infrastructure such as motorcycles and vehicles, communication materials such as airtime and the presence of inspection kits. These were reported to be used in anthrax surveillance and emergency response.

We have local radios. These ones they put in centers. Yes, they’re called local radios. So, it also helps in the campaign. KI 2

the vehicle that was brought in from COVID, that came in from COVID, that vehicle actually became a response vehicle to respond to outbreaks. KI 5

Most of the extension workers from the health department they have motorcycles, most of them, but they are not all, but most of them, they have. KI 4

the VHTs [Village Health Teams], they alert me by either a beep, because when they call, at times they don’t have money, but at least I have airtime. When they beep, I respond. KI 1

We really appreciate ministry gave us the inspection kits. Everything pertaining inspection, whether stamp, whether ink, whether everything, knives and the rest they gave us. So, we are doing inspection and you know the inspection. You have to inspect the animal when it’s still alive, at anti-mortem, when it’s still alive before slaughter. KI 3

#### Communication channels

Participants reported having communication channels for anthrax information flow, surveillance and response done through coordination with human and animal health workers, the presence of social media platforms, opinion leaders, the 6767 alert and the organization of cross-border meetings.

we have 6767 messaging. That one has gone up to village level. And when they send a message, I’m quickly alerted….we have a WhatsApp group where we share problems, where we share information, like thunderstruck animals, we are able to see like the message of yesterday [simulation day]. Almost everybody was able to say and they were calling ***, “you act *** this one said, but you are saying, but where are the animals?” So, that is my strength. KI 1

we use the politicians, give them the information that these doses are given before whenever there is a burial and they sensitize using issues in case there’s a suspected anthrax. That is how the information moves. KI 4

#### Socio-economic activity

The main reported socio-economic activity reported by participants is agriculture and this was mentioned to result in minimal livestock rearing which minimizes the potential of anthrax outbreaks in the district.

because the activities mostly ss agricultural, that is coffee growing, we have maize growing, beans growing and then the other we also have sweet potatoes, cassava, yes, matooke, it is also their bananas. KI 2

### Motivation

#### Beliefs in the ability to respond

Participants had the belief that they were able to respond to an anthrax outbreak due to the presence of competent and trained staff in their district.

When we look at the job description of the local government, in the production department, we recruit diploma holders. In animal production and management. By virtue of then they give them a position to serve government. I believe they are competent enough. KI 2

I believe we can handle because even the facilities were trained, from those various sub counties. They are all equipped and both on the side of the animal and also on the side of the humans. KI 5

#### Fear of anthrax

The fear demonstrated by communities about previous anthrax outbreaks in neighbouring districts was also reported to motivate participants to prepare for anthrax outbreaks.

from what they saw from Buduuda because it was published on the media, on TVs [Televisions] and what. They were scared. First of all, they were scared themselves were the ones who alarmed to show that we have this case around maybe the same anthrax. And even if you sample around, and you talk about anthrax, they see it as a threat. They also know because we have sensitized, they know. KI 3

#### Intentions for disease prevention

Participants demonstrated the presence of plans for zoonotic disease surveillance in preparation for public health emergencies such as anthrax outbreaks through the design of rumour books and training of stakeholders such as the technical staff about anthrax for their protection and competence.

In fact, yesterday [simulation day], the other day, I thought of developing a rumor book I put in LC5’s [Local Council 5] office because everybody goes there. Most of them are from community yeah. So, I thought of developing a rumor book and put there so that whoever feels there is an event, there is a disease, there is an outbreak, the bridge is broken, just report. So, my work would be going there to check what is in the book. KI 1

we train and like now we have got it right now, what we are planning immediately. That is [training] the first thing we have to do. Because we don’t want our staff also to die. If we have a suspect or some confirmed case, and if a person doesn’t know and handle it lightly, we may end up losing staff. So, we first deal with that. KI 3

#### Personal drive/emotions

Participants also reported being self-driven regarding the management of anthrax disease given that it is their responsibility.

I think what I look at is sometimes we have personal zeal, the motive to work because what we always look at is how good we detect fast and what’s the length of our recovery. So, we always look at our recovery. So that, is what we have. Whenever we detect anything, what comes in mind is? How are we going to recover from that? KI 2

### Barriers to readiness for anthrax outbreak response

#### Capabilities

Participants reported the presence of infrastructural challenges in terms of limited IEC materials some of which are in foreign languages insufficient storage capacity of anthrax-suspected samples and no designated burial site for anthrax-suspected carcasses.

last time when there was an outbreak in Buduuda they gave us some [IEC materials] which we supplied…‥The registrar the DHT [District Health Team] have some but they are not enough. They are not always enough. You are saying translating in the local language? Yeah, there were some which were translated and even now because we displayed in health of somebody which has collapsed bleeding, we are showing them that if we don’t see if you see this don’t touch, call a technical person… KI

## We have fridges, yes. not for anthrax samples…Some fridges are designated for those samples. KI 4

We don’t have a specific place for burial. For us in fact, we contain it, we contain it in that same area. We don’t want it to spread and even the people themselves will not allowed to carry it but they will assume you are taking it to another place. KI 2

Regarding regulatory challenges, participants highlighted difficulties they face with unclear objectives of responsible bodies regarding anthrax prevention strategies, mentioned some facilitators of anthrax outbreaks such as frequent border crossings, and emphasized the low implementation of border checks and poor knowledge of communities about anthrax.

the livestock sector we don’t prioritize, generally when I look at the livestock of sector from MAAIF [Ministry of Agriculture, Animal Industry and Fisheries], we don’t have clear objectives. Because as a farmer, I look at productivity, I don’t look at disease. And when you look at the reforms in the agriculture sector, the acquisition of veterinary drugs prevention of the [disease] has really weakened the life of sector. That’s why when it comes to finding there’s nothing. There’s nothing completely because you don’t have clear objectives, because our objective was to improve the heart of the country. We first look at food, we first look at breeds, then we look at disease. KI 2

we have you know, our community goes to Kenya. They buy the small animals, small size, they come and then graze them. But when they mature, they take them back. Because Kenyans tell you they don’t have time to look after those small ones. KI 1

We import bulls from Kenya. We export bulls back to Kenya and central actually, we do not get heifers from Kenya. The Kenyan side, they do not sell heifers. They sell bulls which are like rejects. So, our farmers get the bulls, we improve them with the available percentage breeds we have. KI 2

we don’t have messages. Because we just gamble, you see I’ve been just telling you, I don’t know whether the right thing, but I believe that’s what I know. KI 1

### Opportunities

Participants also highlighted limited resources such as human resources, no resources for the animal health department and surveillance officers to sensitize, no funds for emergency response and no money for timely animal vaccination.

Among the 34 (animal health staff) we have two veterinary surgeons who are at officer level. That means we lack similar veterinary officers, we lack a principal veterinary officer and all those levels. When it comes to management and policy, their job description is different. KI 2

they (veterinary staff) have not done the vaccination part of it. And even the sensitization is not very intense much as they (veterinary staff) are supposed to do it. It’s not very intense. So, they (veterinary staff) wait when the problem arises, that’s when they (veterinary staff) come up. But to them, they (veterinary staff) said they are less funded. They would like to have more training, more meetings with us (human health staff), but they (veterinary staff) are handicapped on the side of funding. KI 1

We have no funds for that. We have no funds to respond, emergence funds to handle the epidemics should they occur. KI 1

Transportation challenges such as few automobiles, sample transportation response challenges and challenges regarding laboratory equipment and vaccination and communication challenges in terms of airtime were also reported.

we only have some few motorcycles, like three for vets. Three or four? But we are 33, I speaking, I don’t have a motorcycle. And the place where I work, even if you reach there, you can even hate life. I wish you go to the field and you see when it rains like this for you cannot move. I’m telling you the truth. KI 3

Second is what is important that also the facilitation of transporting those samples. Because we don’t have like in most cases, we use the Hub system. But now to call the Hub person from because we attached to Tororo. So, calling someone from Tororo to come and pick the sample may take long…. KI 5

And then I don’t have a airtime or fund to promote mainly my messages to promote mainly surveillance. Surveillance is not budgeted before anywhere in the budget in the district. Even human (health). It’s not there, it’s not funded, it’s not allocated. So, there is no airtime. I struggle as me. I use money for the children, for milk, for the children, to buy airtime to make my work move. KI 1

Participants also mentioned challenges regarding insufficient laboratory equipment in addition to the few vaccines received.

Because we have the population hand of around 35 herds of cattle and we share at the national level. They [government] give you 10,000 doses which can’t be reviewed. When we are looking at health, at least we need 70% (vaccination). KI 2

that is the biggest challenge. Biggest challenge. As we talk, both at the district, even at the Mbale regional, regional Hospitals and also even EOC, those materials (laboratory and medical equipment) are not there completely and that’s when there’s an outbreak. We now wonder how we’re going to start. KI 5

### Motivations

Participants emphasized that the limited facilitation for staff activities is a major hindrance to their motivation for anthrax surveillance and response in addition to challenges with funds such as delays in mobilizing funds, minimal contingency funds, salary delays, and no partners to support. Furthermore, participants reported more reactiveness and less proactiveness which is demotivating.

Like I told you, this department is not having any facilitation. So, if I’m to go to the school from here to Tsekululu, you spend around UGX 10,000 [2.7USD] going there and coming back. Even beyond UGX 20,000 [5.39USD] So, you have to put in your money and go there and check. Because the staff who is there will be calling you, who is at the district here. Now, to first take a team to go there, needs transport. When you ask, like, CAO, CAO will tell you, from which sector? Because from your sector you must have a budget must and if the budget is not there, you can’t request what’s not there. KI 3

It (Contingency fund) is there but minimal small actually we depend on support from the region and therefore state much whenever there’s an outbreak they are there, they come here. KI 4

Once they see the outbreak is there, then that’s when they will vaccinate. The officials from the animal side will vaccinate, but as long as the pharmacies, the animal is eating it’s fine. They don’t bother the preventive part of it. KI 1

The poor health communication among departments was also highlighted as a challenge.

But zoonotic diseases here are not so common. Because now you are hearing of anthrax, we had food and mouth the other time and all those were done by veterinary. It’s now that they are bringing us on board, otherwise veterinary which is handling. I remember we had an argument on where to put the samples after they had put their samples in my fridges for humans, we had to throw them out. KI 7

Other commonly mentioned hindrances to anthrax surveillance and response included limited facilities such as slaughter slabs, no animal holding yard at the boarder, no isolation centres at the boarder, poor response practices, illegal cattle trade due to porous borders among others.

we have slaughter slabs around in some areas, but some areas are not having slaughter slabs… The border has no holding yard, even as we have no holding yard. So, that’s the challenge. So, when those ones come, they clear them and they go. KI 3

Whoever brings the animal which is sick in the market, we quarantine it. Yeah, we normally take it to police. For us, we use police. We just call police. They take them to police. KI 3

This is the reality of community. They (community) do the illegal trade there is nothing that you can do. But that one is what happens. KI 2

### Recommendations for improved anthrax outbreak response

Participants suggested enhanced capacity building through sensitisation of communities about anthrax disease, training of staff about their roles regarding anthrax prevention, surveillance and outbreak response, establishment of surveillance guidelines, increment of staff members and use of enforcement procedures.

First is knowledge. Okay. You see when community or when staff has knowledge in management of anthrax, the rest can be innovated. KI 2

And then we need some training on how to respond. Yeah, personally I’ve got, but our team, the animal side, the team in the field, they need that training, they need that knowledge on how to respond. And we need to sensitize our communities more often. KI 7

But if there’s an administrative guideline and policies, that purely surveillance should be this, control of zoonotic diseases should be this it is mandatory. It will really help us. KI 2

And then also increasing the staffing level of laboratory personnel because picking samples may also be a challenge.“ ….”And then sometimes even enforcement, you have to enforce using the police as to come in and enforce. KI 5

Suggested opportunities included the provision of protective equipment for the safe working of staff, the provision of funds for emergency situations, the provision of sample transportation means, the establishment of an animal holding yard at the border, presence of implementing partners among others.

The Red Cross needs to come in to support even the IPs that we have in the region, like the Baylor, we have the city UHA [Uganda Health Activity] that’s Uganda Health activity also needs to come on board. Even CBOs [Community Based Organisations].“ ……” we should think of now equipping the district with emergency materials so that, in case anything happens, we respond.“ …‥”Then also ensuring that the transport means available all the time, to respond in terms of taking samples, taking those health workers to pick the samples from the field. KI 5

That true we need funding from wherever, whether MAAIF or what. What we now need is to strengthen our reporting and surveillance system [20]. Two, also the funding as a sector“ …‥” we need the logistics to support the management. You see, honestly, during that suspected outbreaks, it is God who kept us. But if it was a disease, you don’t get some of us, because we never had the PPEs [Personal Protective Equipment], we had nothing. So, there is having the knowledge, but you don’t have the logistics to manage. KI 2

Participants also suggested monitoring and evaluation through the establishment of anthrax response plans, anthrax vaccination plans, enhancement of slaughter and meat inspections, strengthening of surveillance, ensuring one health meetings and proactiveness through building a surveillance culture.

We need a response plan in place. We need the funds at least where we can begin from before the ministry before Ips [Implementing Partners] can come in.“ ….”we need also to have a plan for vaccinating our animals. we need to vaccinate the animals, and vaccines should be brought nearer to them. Because they vaccinate from that field. You don’t expect somebody from deep on the side of Budduda border to come with an animal up to here. Services should be brought nearer to the people. KI 1

When it comes to surveillance, it is a weak system. Honestly, it is a weak system. It is just matter of reporting, you see, when you report, you need either an enforcement or you need a supplementary funding, isn’t it? But it’s just a matter of send reports, send reports, send reports. What we now need is to strengthen our reporting and surveillance system.“ …”I think we can write proposals and we can attract because we have a lot of productivity here. KI 2

## Discussion

In this study we set out to establish the response capacity for a potential anthrax outbreak in Namisindwa district while following the COM-B models. The generated themes therefore also followed the models in addition to barriers to anthrax response and recommendations for improvement of anthrax outbreak response.

The psychological capabilities of participants mostly were mentioned in their responses through demonstrated knowledge about the disease and response in the face of an anthrax outbreak. The physical capability of participants was also emphasized through their experience and skills in response to similar one health related outbreaks in neighboring districts and zoonotic disease prone areas.

Being knowledgeable is a great indicator of response to an anthrax outbreak and therefore implies that stakeholders in Namisindwa district are capable to respond to an anthrax outbreak. Other studies have also reported knowledge of stakeholders at grassroots especially livestock farmers and other community members about anthrax disease [21, 22] but this simulation study focused on the knowledge at management level among district officials. This study therefore brings an idea of triangulation addition to scientific knowledge regarding the knowledge of stakeholders at district level about their knowledge of zoonotic diseases such as anthrax. Knowledge of district officials can therefore be cascaded to lower levels such as sub-counties and villages of affected areas for efficient and effective prevention, response and management of any anthrax outbreak.

The major physical opportunity presented by stakeholders was their human resource comprised of the district response team and the technical staff at the district and those at the district. Namisindwa district was described by sound leadership and governance where the both the district political and administrative leaders of the district such as the Resident District Commissioner (RDC) and Chief Administrative Officer (CAO) respectively are involved in anthrax surveillance and response. Regarding technical staff, both the animal and human health departments work together through participating in joint zoonotic disease surveillance training programs. Sahoo et al. [23] also found mult-stakeholder engagement for anthrax prevention and control that was implemented through monthly meetings at the district among the human health and veterinary staff. This implies that response to zoonotic diseases calls for multidisciplinary action that considers the staff from human health, environmental health and animal health for effective zoonotic disease prevention, response and management.

Regarding social opportunities, the use of social media platforms increases the flow of communication to various individuals about the onset of anthrax outbreak. However, it is important to note that the reliability of information received through social media platforms is low among district officials and hence barely triggers response to anthrax outbreaks. Anthrax information on social media however triggers response from stakeholders at central bodies such Ministry of Health, MAAIF and Emergency Operations Centres as rumors in need of verification, and this has also been found in other studies [24].

Automatic motivation was mainly demonstrated as a challenge of limited reinforcements in form of poor facilitation and resources for conduction of anthrax prevention related activities. Omodo et al. [25] also found lack of resources and weak guidelines especially in the veterinary department as a challenge for conducting anthrax response activities. This implies that the veterinary departments in the anthrax prone districts are indeed low on resources and funds for anthrax surveillance and response activities.

Although human and animal health aspects of anthrax outbreak response were identified in this study, the environmental health aspect was barely reflected in the study therefore the One Health approach was incomplete. Although stakeholders recognized the need for working as a one health team, we understood that each sector conducts their duties in isolation regarding activities and budgets which brings conflicting and overlapping activities.

The participants seemed to think that anthrax was mainly an animal disease which is recognized in public after spill over among the humans [25] due to their cultural beliefs and practices [22, 26]. This implies that surveillance in the environmental and animal health sectors are barely or ineffectively conducted to prevent anthrax before reaching the humans.

The study team recommends the district to design a contingency plan for a possible anthrax outbreak and increase knowledge about anthrax among community members to increase their alertness about any anthrax related rumors. The district leadership should consider following the internationally recommended carcass disposal methods to prevent human and animal anthrax [27] including incineration as the ideal solution. The district should therefore establish incinerating centres for safe disposal of suspected anthrax carcasses. The district should also stock formalin for pouring on the carcass and the immediate environment or location of sick and/or death of the animal. Where the above are unavailable, the district should consider deep burial of the carcass at least 6 ft in the ground in a location with minimal chances of earth disturbance.

Prevention mechanisms particularly animal vaccination is vital in preventing future outbreaks. Given the fact that anthrax is a private good disease in Uganda, therefore needs individual farmers efforts for vaccination to effect [28]. However, farmers in low resource settings like Uganda face financial challenges to individual vaccinate their animals against anthrax. More funding is therefore required not only for anthrax preparedness, resilience and response of the district, but also to aid farmers with vaccination. The district leadership should also ensure easy access to the anthrax animal vaccine and educate farmers on the relevance of this vaccination in prevention of future outbreaks. Future researchers should consider use of other models to discover more themes after simulation of anthrax outbreaks in anthrax prone areas.

This study has a number of limitations; the interviewed stakeholders did not include all team members from the different sectors of human, environmental and animal health from the district. The responses of the stakeholders cannot be replicated given that this was a qualitative study; however, the study elicited the stakeholders provide information that may not have been gotten in case a structured questionnaire was used for data collection.

## Conclusions

Generally, the district was partially ready for the next anthrax outbreak. The district was resourced in terms of human resource but lacked adequate funds for animal, environmental and human surveillance activities for anthrax and related response. The district technical staff had the knowledge required to respond to anthrax outbreak but lacks adequate funds for animal, environmental and human surveillance for anthrax and related response. We think that our study findings are generalizable in similar settings and therefore calls for implementation of such periodic evaluations to help leverage the strong areas and improve other aspects. Anthrax being a growing threat in region, there should pro-active efforts in prevention specifically we recommend vaccination of livestock and further research for human vaccines.

## Supporting information

Walekhwa et al. supplementary material Walekhwa et al. supplementary material

## Data Availability

All the datasets and other related resources associated with this manuscript are available and can be accessed from the corresponding author after submitting a justifiable request to Abel W. Walekhwa, wabelwilson@gmail.com

## Supplementary material

For supplementary material accompanying this paper visit http://dx.doi.org/10.1017/S0950268824001493. click here to view supplementary material
